# MicroRNA396-mediated alteration in plant development and salinity stress response in creeping bentgrass

**DOI:** 10.1038/s41438-019-0130-x

**Published:** 2019-05-01

**Authors:** Shuangrong Yuan, Junming Zhao, Zhigang Li, Qian Hu, Ning Yuan, Man Zhou, Xiaoxia Xia, Rooksie Noorai, Christopher Saski, Shigui Li, Hong Luo

**Affiliations:** 1 Department of Genetics and Biochemistry, Clemson University, 110 Biosystems Research Complex, Clemson, SC 29634-0318 USA; 2 State Key Laboratory of Hybrid Rice, Rice Research Institute, Sichuan Agricultural University, 611130 Chengdu, Sichuan China

**Keywords:** Salt, Plant morphogenesis, Agricultural genetics

## Abstract

The conserved microRNA396 (miR396) is involved in plant growth, development, and abiotic stress response in multiple plant species through regulating its targets, *Growth Regulating Factor* (*GRF*) transcription factor genes. However, the role of miR396 has not yet been characterized in perennial monocot species. In addition, the molecular mechanism of miR396-mediated abiotic stress response remains unclear. To elucidate the role of miR396 in perennial monocot species, we generated transgenic creeping bentgrass (*Agrostis stolonifera*) overexpressing *Osa-miR396c*, a rice miRNA396 gene. Transgenic plants exhibited altered development, including less shoot and root biomass, shorter internodes, smaller leaf area, fewer leaf veins, and epidermis cells per unit area than those of WT controls. In addition, transgenics showed enhanced salt tolerance associated with improved water retention, increased chlorophyll content, cell membrane integrity, and Na^+^ exclusion during high salinity exposure. Four potential targets of miR396 were identified in creeping bentgrass and up-regulated in response to salt stress. RNA-seq analysis indicates that miR396-mediated salt stress tolerance requires the coordination of stress-related functional proteins (antioxidant enzymes and Na^+^/H^+^ antiporter) and regulatory proteins (transcription factors and protein kinases). This study establishes a miR396-associated molecular pathway to connect the upstream regulatory and downstream functional elements, and provides insight into the miRNA-mediated regulatory networks.

## Salt tolerance: How plants handle high (salt) stress situations

A small molecule, miR396, plays a big role in how plants respond to salt stress, a growing constraint on global crop yield. Salt stress prevents plants from absorbing nutrients, causes oxidative stress, and destroys key cellular machinery; some plants cope better than others. Hong Luo at Clemson University in South Carolina, USA and coworkers investigated how creeping bentgrass handles high salinity, focusing on the microRNA miR396, previously implicated in salt tolerance. Artificially elevating miR396 improved bentgrass’ salt tolerance. Further investigation showed that miR396 triggered increases in proteins that pump excess salt out of cells, others that prevent water loss, and antioxidant production. miR396 was found to be a master regulator that orchestrates many lines of defense against excess salt. Understanding how these pathways are activated and integrated could help in breeding more salt-tolerant crops.

## Introduction

Soil salinity is a major constraint for crop productivity. High Na^+^ content during salt stress results in ionic stress due to the disturbed intracellular ion homeostasi^1^. Excess Na^+^ negatively affects K^+^ uptake because of their similar chemical properties, and thereby leads to the inhibition of many K^+^-dependent biological processes, such as protein synthesis, enzymatic reactions, and photosynthesis. To alleviate ionic stress and maintain a high K^+^:Na^+^ ratio, plants have evolved adaptive strategies of cytosolic Na^+^ exclusion and vacuolar Na^+^ sequestration^2^. Currently, several classes of Na^+^ transporters have been identified to play central roles in these adaptive strategies during high salinity exposure^3,4^. The best-characterized Na^+^ transporters, which alleviate excess Na^+^ through compartmentalizing Na^+^ into the vacuoles are NHX transporters via mediating intracellular Na^+^/H^+^ and K^+^/H^+^ antiport^5^. Studies in various plant species show that overexpression of NHX genes confers enhanced high salinity tolerance and elevated relative Na^+^ content in plant tissues^6,7^. Besides Na^+^ sequestration, the mechanism of Na^+^ exclusion has been well elucidated through characterization of salinity overly sensitive 1 (SOS1) transporters, which mediate Na^+^/H^+^ antiport across the plasma membrane^8^. The SOS signaling pathway in response to salt stress has been proposed in *Arabidopsis*, in which the calcium-binding protein AtSOS3 activates the kinase activity of AtSOS2 to further activate AtSOS1 through the direct phosphorylation^9,10^. Overexpression of *AtSOS1* has been shown to lead to enhanced salt tolerance in transgenic (TG) *Arabidopsis*^11^.

Salt stress also induces osmotic stress due to the accumulation of Na^+^ in the apoplast, thereby elevating osmotic gradient between inside and outside of the cells. In response to osmotic stress, certain organic solutes called osmoprotectants (such as proline, sugars, mannitol, etc.) are produced in the cytoplasm to maintain cell turgor pressure^12^. A previous study indicated that TG rice accumulating sugar trehalose exhibits enhanced abiotic stress tolerance^13^.

In addition, salt stress leads to enhanced production of reactive oxygen species (ROS), such as superoxide, hydrogen peroxide, hydroxyl radical, and singlet oxygen, which trigger cell oxidative damage and ultimately cell death^14^. Antioxidant enzymes, such as superoxide dismutase, catalase, guaiacol peroxidase, ascorbate peroxidase, dehydroascorbate reductase, and glutathione reductase, play an essential role in scavenging the overproduced ROS. Overexpression of genes encoding these antioxidants confers enhanced tolerance to salt and other abiotic stresses in a variety of plant species^15,16^.

Besides these functional proteins, regulatory roles of transcription factors (TFs), protein kinases, and phosphatases have also been well documented in the plant response to salt stress. Recently, the regulatory role of microRNAs (miRNAs) in plant response to salt and other environmental stresses has been gradually revealed. For example, TG *Arabidopsis* with higher expression levels of miR408 exhibits enhanced tolerance to salt, cold, and oxidative stresses, but reduced tolerance to drought and osmotic stresses^17^. MiR408-mediated plant abiotic stress response is associated with enhanced cellular antioxidant capacity and reduced ROS production^17^. Another recent study shows that constitutive expression of *Osa-miR528* confers enhanced salt and nitrogen-deficiency tolerance in TG creeping bentgrass, which is associated with up-regulation of K^+^ transporter gene *HAK5*, the increased activities of nitrite reductase and antioxidant enzymes, altered expression of other stress-related TF and small RNAs, and the repression of its direct targets^18^. These results strongly suggest that miRNAs coordinate multiple stress-responsive pathways to cope with abiotic stress.

MiR396 is a conserved miRNA, and presents in both monocots and dicots. It performs post-transcriptional gene regulation through repressing its targets, *Growth Regulating Factors* (*GRFs*). The role of miR396 in plant growth and development has been well characterized. Morphologically, TG plants constitutively expressing miR396 display shorter plants and narrower leaves than WT controls in *Arabidopsis* and tobacco because of reduced cell numbers in leaf^19–21^. A similar phenotype was observed in *atgrf1 atgrf2 atgrf3* triple mutants^19^. In addition, TG tobacco overexpressing miR396 showed cotyledon fusion and lack of a shoot apical meristem^22^. A recent study showed that *AtGRF5* participates in the control of leaf senescence^23^. TG *Arabidopsis* overexpressing miR396 exhibits an early senescence phenotype^23^. MiR396 is also involved in plant response to various abiotic stresses. *At-miR396* was induced under UV-B radiation resulting in inhibited cell division in proliferating tissues^24^. Expression of tomato *miR396a* (*Sp-miR396*) is up-regulated under salt and drought stresses^25,26^. TG tobacco overexpressing *Sp-miR396a* exhibits enhanced tolerance to salt, drought, and cold due to the improved osmoregulation and decreased accumulation of ROS^26^. In contrast, constitutive expression of *Osa-miR396c* in TG rice and *Arabidopsis* results in reduced salt and alkali stress tolerance in comparison with wild-type (WT) plants^27^. The opposite responses to salt stress in different plant species suggest a species-specific function of miR396. Currently, the role of miR396 has not been elucidated in perennial monocot species. In addition, the underlying molecular mechanisms of miR396-mediated plant resistance to salt and other environmental stresses remain unclear.

In this study, we generated TG creeping bentgrass plants constitutively expressing rice miR396 to investigate the role of miR396 in plant development and response to salt stress in this perennial monocot species. MiR396 TG plants display altered leaf morphology and tillering, and improved salt stress tolerance in comparison with WT controls. Furthermore, genome-wide analysis in miR396 TG plants vs. WT controls elucidates the possible regulatory pathway for miR396-mediated salt or other environmental stress responses, which provides insight into the central role of miR396 in the regulatory network.

## Materials and methods

### Generation of TGs overexpressing *MiR396*

A 510 bp DNA fragment of *Os-miR396c* gene (GenBank: AK062523.1) containing pre-miR396c stem-loop structure was amplified from rice cDNA. The cDNAs were then cloned into the pGEM-T Easy vector (Promega, Madison, WI). T Easy-cDNA with the correct sequence was digested and recombined into the binary vector pZH01^28^ generating an overexpression gene construct, which was then introduced into creeping bentgrass cultivar ‘Penn A-4’ (supplied by HybriGene) via *Agrobacterium tumefaciens* strain LBA4404-mediated plant transformation. Creeping bentgrass transformation using mature seed-initiated embryonic callus was as described previously^29,30^.

### Plant growth and salt stress treatment

WT and the regenerated TG creeping bentgrass were clonally propagated and grown in cone-tainers (4.0 × 20.3 cm, Dillen Products) filled with pure silicon sand. The plants were fertilized every other day with 0.2 g/L 20:10:20 water-soluble fertilizer (Peat-Lite Special; The Scotts Company) and maintained in a growth room with light regime (14-h of light/ 10-h of dark). Temperatures in the growth room were 25 °C during the light period and 17 °C during the dark period with 350–450 μmol m^−2^ s^−1^ light intensity. WT and TG plants were propagated at the same time and from the same amount of tillers to ensure that they were at the same developmental stage for morphological analysis and salt stress treatment.

Salt stress treatment and leaf sample collection for gene expression analysis were performed as described previously with modifications^18^. In this study, the concentration of NaCl solution was 250 mM. The duration for salt stress treatment was 8 days followed by a 10-day recovery.

### Measurement of mineral content, leaf RWC, chlorophyll, and proline content

WT and TG plant leaf tissues were collected before and after 8-day, 250 mM NaCl treatments for the measurement of Na^+^ and K^+^ relative contents, leaf RWC, EL, total chlorophyll content, and proline content using previously published protocols^31^.

### Histology analysis

Histological analysis of the leaf and stem cross sections was performed as described previously with modifications^18^. In this study, sections were stained using Toluidine Blue or Safranin. For leaf epidermis cells observation, clear nail polish was applied to the leaf upper epidermis of the representative WT and TG plants. After drying completely, the films were peeled off and observed under microscope (MEIJI EMZ-5TR).

### Plant RNA isolation and expression analysis

Plant total RNA was isolated from 100 mg of the first and second topmost fully expanded leaves of each tiller. RNA isolation, semi-quantitative RT-PCR and stem-loop RT-qPCR analyses were performed according to previously published protocol^18^. *AsUBQ5* (JX570760) was used as an endogenous control. All primers used for sequence amplification, quantitative, and semi-quantitative RT-PCR analysis are listed in supplementary Table 1.

### cDNA library preparation and Illumina sequencing, differential expression and GO enrichment analyses

Total RNA of shoots from non-stressed WT and TG plants were extracted and purified using the RNeasy Plant Mini Kit (Qiagen, Germantown, MD). cDNA libraries were constructed using TruSeq RNA Library Preparation v2 (Illumina Inc., San Diego, CA) according to the manufacturer’s protocol. Paired-end sequencing of each library was performed using the HiSeq 2000 (Illumina Technologies) platform following the manufacturer’s instructions (101-bp paired-end reads).

The differential gene expression analysis was performed using R 3.2.0 (https://www.r-project.org) and the Bioconductor package edgeR with FDR-corrected *P*-value cut-off of <0.05. A MDS plot was generated using edgeR to show the separation between WT and TG samples and consistency between replicates. A volcano plot was created using edgeR to plot log_2_ FC and the –log_10_ *P*-value in TG vs. WT. The heatmaps showing expression profiles between WT and TG samples were generated based on the log_2_-transformed count values using R’s pHeatmap.2 package (https://cran.r-project.org/web/packages/pheatmap/index.html).

To gain information on the over-represented functional categories, GO-enrichment analysis was performed. Since there is no GO annotation available for creeping bentgrass transcripts, putative GO terms were assigned using NCBI-blast^+^ and Blast2GO 2.8^32,33^.

## Results

### MiR396 is regulated by salt stress

Previous studies showed that miR396 responds to salt stress in various plant species, displaying differential expression profiles. For example, miR396 is down-regulated under salt stress in rice, *Spartina alternaiflora*, *Populus cathayana*, and *Salix matsudanabut* Koidz, but up-regulated in tomato^26,27,34,35^. To investigate the role of miR396 in response to salt stress in a perennial grass species, creeping bentgrass, we set to first study how miR396 responds to salt stress through analyzing its expression profile. Stem-loop RT-qPCR analysis showed that miR396c was significantly up-regulated upon salt treatment (Fig. S1). Although declined 6 h after treatment, it remained noticeably elevated compared to 0 h control (Fig. S1). The result suggests that miR396 might act as a positive regulator in plant salt stress response.

### Generation of TG creeping bentgrass constitutively expressing *Osa-miR396c*

After confirming that miR396c responds to salt stress, we generated TG creeping bentgrass constitutively expressing a rice gene, *Osa-miR396c* to further study the role of miR396 in plant adaption to salt stress (Fig. S2a). The constitutive expression construct of miR396 was introduced into wild type (WT) plants via *A. tumefaciens*-mediated transformation. The selectable marker gene, *Hyg* conferring hygromycin resistance was amplified from the genomic DNA of regenerated TG plants and WT controls for TG event selection (Fig. S2b). The expression levels of *pri-miR396c* and mature *miR396c* were then compared between WT and TG plants to determine whether the rice pri-miR396c was successfully integrated into the genome of creeping bentgrass, transcribed, and properly processed (Fig. S2, c and d). We generated a total of 32 individual TG events, which exhibit small leaf area and slow growth rate in comparison with WT controls.

### TG creeping bentgrass overexpressing *MiR396c* exhibits altered plant development

**Fig. 1:**
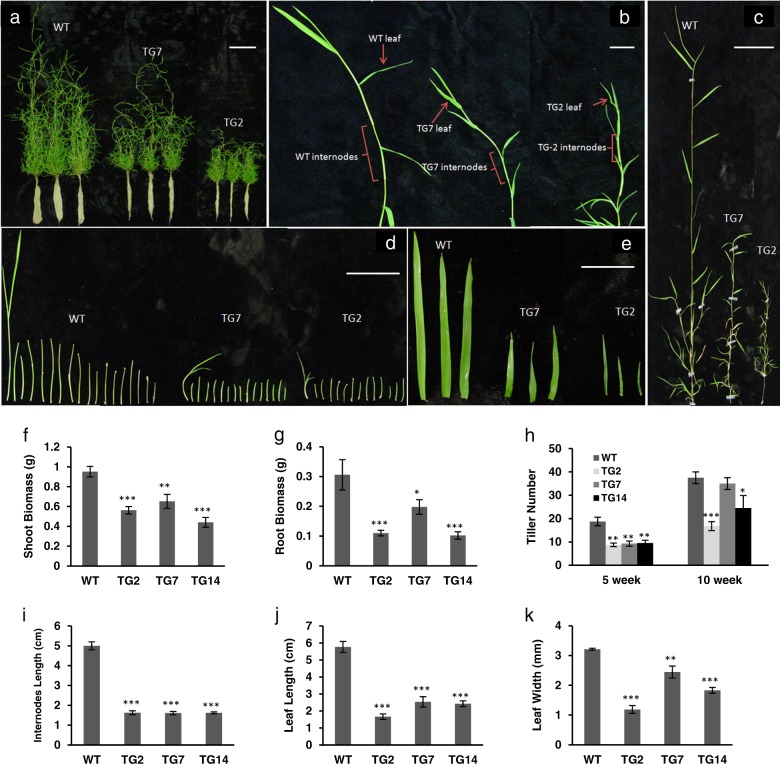
Plant tillering and development. **a** Ten-week-old WT and TG plants both initiating from a single tiller. Scale bar, 10 cm. **b** Representative leaves and internodes of WT and TG plants after initiation from single tillers. Scale bar, 2 cm. **c** Close up of the longest tillers from WT and TG plants. Scale bar, 5 cm. **d** All internodes from the representative longest tiller were sliced from top to bottom and arranged from left to right. Scale bar, 5 cm. **e** Three of the topmost fully developed leaves from the representative tillers of WT and TG plants. Scale bar, 2 cm. **f** Shoot and **g** root dry weight of WT and TG plants at 10 weeks after initiation from a single tiller (*n* = 4). **h** Tiller number in WT and TG plants at 5 and 10 weeks after initiation from a single tiller (*n* = 5). **i** Average length of the topmost eight internodes from WT and TG tillers (*n* = 6). **j** Leaf length and **k** leaf width from the representative WT and three transgenic lines (*n* = 3). Data are shown as means, and error bars represent standard error. A significant difference between WT and each TG line was indicated with asterisks (*, **, or ***) at *P* *<* 0.05, 0.01, or 0.001 by Student’s *t*-test

To study if miR396 is implicated in plant development of perennial grass species, 10-week-old WT and TG plants each initiated from a single tiller were compared. As shown in Fig. 1a, TG plants produced smaller shoots and fewer roots than those of WT controls. As a result, TG plants had significantly reduced biomass accumulation in both shoot and root compared to WT controls (Fig. 1f, g). Further analysis of tiller growth indicates that the reduced shoot biomass in TG plants compared to WT controls is associated with shorter tiller length and/or lower tiller number than WT controls (Fig. 1c, h). The average length of the internodes from the longest TG tillers is significantly reduced compared with that of the WT tillers (Fig. 1b, d, and i), resulting in reduced tiller length in TGs. When comparing leaf morphology between WT and TG plants, TG leaves are narrower and shorter than WT leaves (Fig. 1b, e, j, and k), similar to the morphology changes observed in TG *Arabidopsis* and tobacco constitutively expressing miR396^11,21^. The result suggests that the role of miR396 in controlling leaf development is conserved between monocots and dicots.

**Fig. 2:**
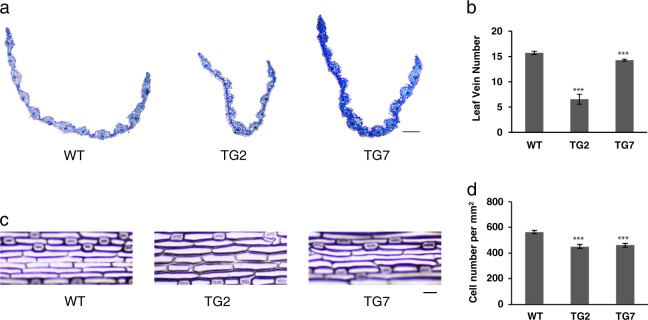
Histological analysis of leaf and stem of WT and TG plants. **a** Cross-section images of WT and TG leaves. Scale bar, 100 µm. **b** Statistical analysis of leaf vein number between representative WT and TG plants (*n* = 5). **c** The representative leaf epidermis of WT and two TG lines. Scale bar, 50 µm. **d** The number of leaf epidermis cells between WT and two TG lines (*n* = 5). Data are shown as means with standard error. Asterisks (***) indicate a significant difference between WT and each TG line at *P* *<* 0.001 by Student’s *t*-test

Previous study indicated that miR396 is involved in cell proliferation^21^. To study the impact of miR396 on plant development at cellular level, we cross-sectioned leaves and stems in WT and TG plants followed by histological analysis. As shown in Fig. 2a, c, two TG lines have significantly fewer leaf veins than WT controls, resulting in narrower leaves. TG2 plants with FL phenotype displayed reduced stem diameter and a reduced number of vascular bundles, while TG7 plants with the WL phenotype did not show a significant difference from WT controls (Fig. 2b, d, and e). To investigate if the smaller leaf size in TG plants is attributed to the reduced cell proliferation, we compared the number of leaf epidermis cells in WT and TG plants. The result shows that the leaf epidermis cells in TG plants are significantly reduced compared to WT controls (Fig. 2f, g), implying that miR396 is a negative regulator in cell proliferation.

### MiR396 TG plants exhibit enhanced salt tolerance associated with improved water retention and cell membrane integrity, increased chlorophyll contents, and decreased proline contents under salt stress

**Fig. 3:**
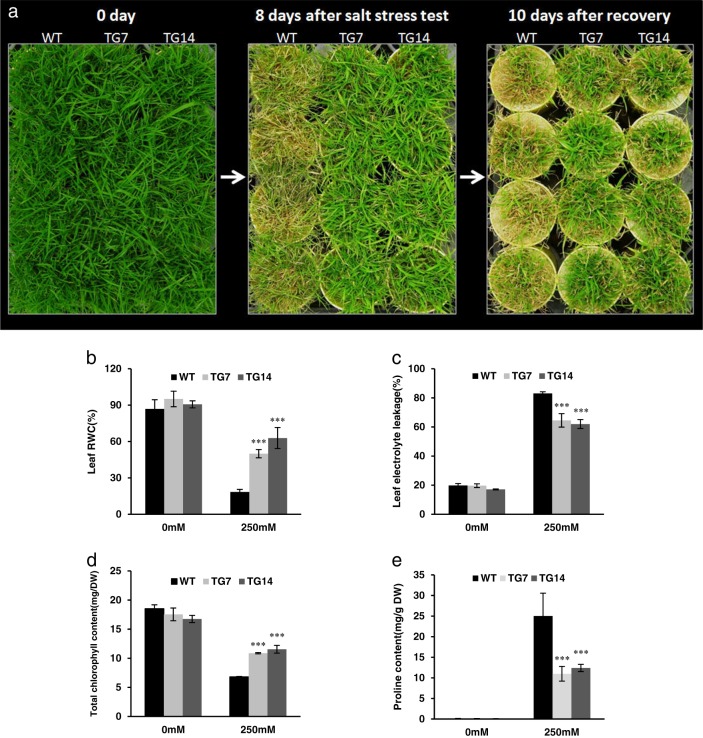
Responses of WT plants and TG lines under salt stress test. **a** WT and TG plants were subjected to 250 mM NaCl treatment for 8 days followed by a 10-day recovery. **b** Leaf RWC, **c** leaf electrolyte leak, **d** total chlorophyll content, and **e** proline content were measured in WT and TG leaves at 0 day and 8 days of salt stress treatment. DW dry weight. Data are shown as means (*n* = 4) with standard error. Asterisks (***) indicate a significant difference between WT and each TG line at *P* < 0.001 by Student’s *t*-test

To study the role of miR396 in plant response to salt stress in creeping bentgrass, we chose to use two low transgene expressers, TG7 and TG14 with moderately altered morphology for analysis. Both WT and TG plants initiating from the same number of tillers were mowed to the same height prior to salinity exposure. Plants were then treated by 250 mM NaCl for 8 days followed by a 10-day recovery. As shown in Fig. 5a, upon NaCl treatment and during plant recovery, most of the WT leaves senesced and turned yellow, whereas the TG leaves remained green with little damage (Fig. 3a), indicating that overexpression of miR396c leads to enhanced salt tolerance in TG plants.

To further investigate what causes the enhanced salt tolerance in TG plants at physiological level, we analyzed leaf relative water content (RWC), leaf electrolyte leakage (EL), total chlorophyll content, and proline content in WT and TG plants before and 8 days after 250 mM NaCl treatment. Before the treatment, WT plants and two TG lines have similar RWC (Fig. 3b). Upon exposure to salt stress, although the RWC of both WT and TG plants declined, the decline in TG plants was significantly less pronounced than that of WT controls (Fig. 3b), indicating that TG plants have a better capacity of water retention under salt stress. The EL of WT and TG plants was similar under normal growth conditions, and then increased dramatically after NaCl treatment (Fig. 3c). However, EL in TG plants was significantly lower than that in WT controls (Fig. 3c), suggesting that TG plants have improved ability to maintain cell membrane integrity during salt exposure. Salt stress leads to chlorophyll breakdown and induces leaf yellowing. In this study, total chlorophyll content of WT and TG plants was similar before the salt stress test, but declined after salt stress exposure (Fig. 3d). However, the decline in WT controls was significantly more pronounced than that in TG plants (Fig. 3d), which may contribute to the robustness of photosynthetic system. Proline is accumulated under salinity conditions and acts as an osmolyte to protect plants from salt stress-induced toxic oxygen derivatives. Proline contents in WT and miR396 TG creeping bentgrass plants were similar under normal growth conditions, but increased dramatically after NaCl application (Fig. 3e). Interestingly, proline content in two TG lines was significantly lower than that in WT controls (Fig. 3e). Currently, the nexus between proline accumulation and abiotic stress tolerance remains controversial^36^. Additionally, previous study indicates that proline homeostasis is more critical than proline accumulation for the maintenance of plant growth and development under stress conditions^37^. Thus, it is likely that TG plants have improved capacity to maintain proline homeostasis and thereby contribute to the enhanced salt stress tolerance. It is also likely that the less salt-elicited leaf damage in TG plants triggers less accumulation of proline than WT controls.

### Enhanced salt tolerance in TG plants is associated with reduced Na^+^ uptake

**Fig. 4:**
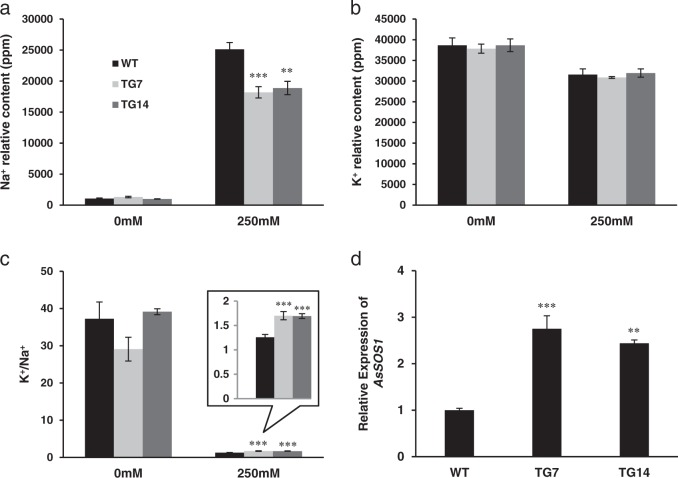
Na^+^ and K^+^ relative contents. **a** Na^+^ relative content and **b** K^+^ relative content were measured before and after salt stress treatment in the leaf tissue of WT and two TG lines. **c** K^+^:Na^+^ ratio before and after salt test in WT and two TG events. **d** Quantitative RT-PCR analysis of the expression levels of *AsSOS1* in WT and two TG lines under normal growth conditions. *AsUBQ5* was used as the reference gene. Data are shown as means (*n* = 3) with standard deviation. Asterisk(s) indicates significant differences between WT and each TG line at *P *<* 0.05; **P *<* 0.01; ***P *<* 0.001 by Student’s *t*-test

The adaptive mechanisms of plants in response to salt stress include exclusion of Na^+^ and/or compartmentalization of Na^+^ into the vacuole. To elucidate the mechanism of miR396-mediated salt tolerance, we compared the Na^+^ content between WT and TG plants under normal and salt stress conditions. The result shows that the two TG lines accumulate significantly less Na^+^ than WT controls under salt stress conditions (Fig. 4a), suggesting that the enhanced salt tolerance in miR396 TGs may be attributed to the exclusion of Na^+^.

High concentration of Na^+^ during salt stress leads to limited K^+^ uptake and it might substitute for K^+^ in some K^+^-dependent protein interactions within plant cells. Thus, the ability of K^+^ retention and a high K^+^:Na^+^ ratio is important for salt tolerance. In this study, the K^+^ relative content is similar in WT and TG plants under both normal and stressed conditions (Fig. 4b), while the K^+^:Na^+^ ratio is significantly elevated in TG plants under salinity conditions (Fig. 4c), which might contribute to the enhanced salt tolerance.

### MiR396 is involved in the regulation of Na^+^ transporter *SOS1*

The relatively low Na^+^ content in TG plants under salt stress conditions might result from the enhanced ability to extrude Na^+^ from the cytosol across the plasma membrane. SOS1 is the best-known Na^+^ transporter, which functions in Na^+^ exclusion. To reveal the molecular mechanism of miR396-mediated salt tolerance, we cloned a partial sequence of *SOS1* in creeping bentgrass and analyzed the expression levels of *AsSOS1* in WT and two TG lines. Quantitative RT-PCR analysis shows that *AsSOS1* exhibits elevated expression in TG plants compared with WT controls (Fig. 4d), suggesting that miR396 mediates salt tolerance through Na^+^ excluding mechanism.

### Identification of MiR396c putative targets and their responses to salt stress

**Fig. 5:**
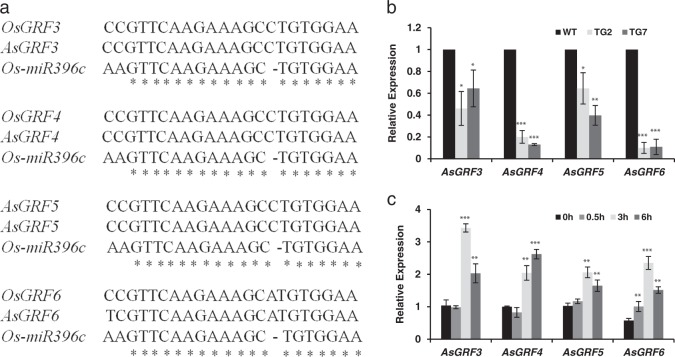
Identification of putative miR396c targets in creeping bentgrass. **a** A comparison of miR396c target sites in the putative targets *AsGRF3*, *AsGRF4*, *AsGRF5*, and *AsGRF6* between rice and creeping bentgrass. Asterisks indicate the identical RNA sequences. **b** Quantitative RT-PCR analysis of *AsGRF3*, *AsGRF4*, *AsGRF5*, and *AsGRF6* expression in WT and TG plants. **c** Expression profiles of miR396c putative targets in response to salt stress. Transcript levels of *AsGRF3*, *AsGRF4*, *AsGRF5*, and *AsGRF6* were analyzed at 0, 1.5, 3, and 6 h after 250 mM NaCl treatment using real-time RT-PCR. *AsUBQ5* was used as an endogenous control. Data are presented as means of three technical replicates, and error bars represent ± SE. Asterisk(s) indicates a significant difference of expression levels between WT and each transgenic line at *P < 0.05; **P *<* 0.01; ***P *<* 0.001 by Student’s *t*-test

To elucidate the molecular mechanism of miR396-mediated plant development and salt tolerance, we sought to identify the putative targets of miR396 in creeping bentgrass. It has been reported that the targets of miR396 encode the TFs of the GRF family. In *Arabidopsis*, *GRF* gene family contains nine members (*AtGRF1*–*9*), seven of which have miR396-binding site except *AtGRF5* and *AtGRF6*^38^. In rice, 12 members (*OsGRF1*–*12*) were identified, 10 of which contain miR396c target site except *OsGRF11* and *OsGRF12*^38^. Expression analysis of *OsGRFs* in WT rice and TG rice overexpressing *Osa-miR396d* indicates that 9 out of 12 *OsGRFs* are repressed in TG plants, which are *OsGRF1*-*8* and *OsGRF10*^39^. In this study, four *GRFs* (*AsGRF3*–*6*) were successfully cloned in creeping bentgrass based on the sequence of *GRF* homologs in rice and *Brachypodium*. MiR396c target sites in *AsGRF3*–*6* were identified and compared with the target sites of rice *OsGRF3*–*6* as shown in Fig. 5a. The expression of these four genes was repressed in TG2 and TG7 lines compared with that in WT controls (Fig. 5b), suggesting that they are the putative targets of miR396c in creeping bentgrass. GRFs play an essential role in plant leaf, stem, and root growth and development^40^. Therefore, it is plausible to speculate that the repressed expression of the *AsGRFs* might contribute to the altered plant development in miR396 TG creeping bentgrass.

Next, we analyzed the expression patterns of *AsGRFs* under salt conditions to investigate how the miR396-GRF pathway is implicated in plant salt tolerance. Quantitative RT-PCR analysis shows that expression levels of *AsGRF3*–*6* are all up-regulated in response to 250 mM NaCl treatment (Fig. 5c).

### Genome-wide analysis of gene expression in relation to miR396-mediated plant development and salt stress tolerance

MiRNAs serve as master regulators to integrate different regulatory pathways to control plant development and plant response to environmental stress. To gain insight into the miR396-mediated regulatory network, we performed RNA-seq analysis to study the differentially expressed genes (DEGs) in miR396 TG plants vs. WT controls. Shoots RNA of non-stressed WT and TG were isolated for cDNA library preparation. Illumina sequencing generated 4,444,691 contigs, which were further assembled into 82,819 unigenes with an average size of 995.5 bp. The reproducibility of RNA-seq analysis was confirmed by multidimensional scaling (MDS) plot (Fig S3a), which shows the expected consistency between two biological replicates of WT and TG samples, respectively. A volcano plot shows the distribution of Log_2_-fold changes (FC) of 17,338 unigenes at false discovery rate (FDR) corrected *p*-value < 0.05 (Fig S3b and c). Among the differentially expressed transcripts (Log_2_ FC > 2 or <−2), 584 are up-regulated and 1027 are down-regulated in TG plants vs. WT controls.

### Gene ontology (GO)-enrichment analysis

To identify the putative biological processes of DEGs in TG vs. WT plants, we performed GO-enrichment analysis. In this study, 9 and 12 GO terms in the biological process category were significantly enriched (over-represented *P-*value < 0.05) in the up-regulated and down-regulated genes (log_2_ FC > 2 or log_2_ FC < −2), respectively (Fig. S4). Among them, significantly enriched GO terms of ‘oxidation–reduction process’, ‘response to oxidative stress’, and ‘hydrogen peroxide catabolic process’ suggest that miR396 might be involved in the oxidation–reduction process. Salt stress results in ROS accumulation in plant cells and creates oxidative environment and imbalanced redox state. Oxidation–reduction process plays a fundamental role in regulating redox homeostasis and might contribute to the enhanced salt tolerance in miR396 TG plants. Besides the stress tolerance-related GO terms, GO terms of ‘DNA replication’, ‘cell division’, ‘regulation of cell cycle’, and ‘mitotic nuclear division’ were all significantly enriched in the down-regulated genes (Fig. S4b), indicating that TG plants might have less cell division and fewer cell numbers, consistent with what we observe in TG leaves.

### Differential expression of leaf development-related and stress response-related genes

In addition to identify the overrepresented GO terms in the whole DEGs of TG vs. WT dataset, we also analyzed the leaf development-related and stress response-related DEGs. Significantly enriched GO terms (over-represented *P*-valueP < 0.05) in the DEGs (log_2_ FC > 1 or log_2_ FC < −1, FDR-corrected *P*-value < 0.05) were identified, in which the corresponding genes related to leaf development and environmental stress response were chosen for generating a heatmap. As shown in Fig. S5, 201 DEGs had enriched GO terms of ‘DNA replication’, ‘cell differentiation’, ‘cell division’, ‘leaf development’, etc. In addition, 603 DEGs had enriched GO terms related to biotic and abiotic stress responses, including response to cold, salt, heat, drought, wounding, light, heavy metal, nutrient deficiency, bacterium, virus, and fungus (Fig. S5). The result demonstrates that miR396 affects a variety of biological processes.

### Validation of expression profiles of candidate genes

**Fig. 6:**
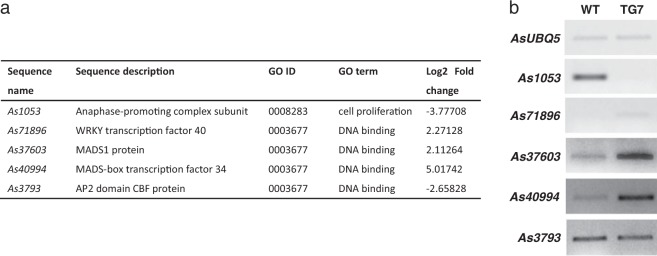
Identification of putative miR396c targets in creeping bentgrass. **a** A table of sequence information of DEGs, *As1053*, *As71896*, *As37603*, *As40994*, and *As3793*. **b** Semi-quantitative RT-PCR analysis of the expression patterns of DEGs in WT and TG plants under normal growth conditions. *AsUBQ5* was used as an endogens control

To validate the expression profiles of DEGs in RNA-seq data, we analyzed expression levels of candidate genes involved in cell reproduction and environmental stress response (Fig. 6). *As1053* is predicted to encode a subunit of anaphase-promoting complex involved in cell reproduction. RT-PCR analysis shows repressed expression in two TG lines compared with that in WT controls (Fig. 6), in agreement with RNA-seq result. Also consistent with the RNA-seq data, a predicted WRKY40 transcription factor gene, *As71896* and two MADS-box genes, *As37603* and *As40994* are up-regulated in TG plants in comparison with WT controls (Fig. 6). Furthermore, *As3793*, a predicted AP2 transcription factor gene, is down-regulated in both RT-PCR and RNA-seq results (Fig. 6).

## Discussion

### MiR396-mediated plant development during vegetative growth

The *miR396*-*GRF* regulatory pathway plays a key role in leaf development. Overexpression of *At-miR396a* leads to smaller and narrower leaves through suppressing *GRF* genes in TG tobacco^20^. In this study, TG creeping bentgrass overexpressing *Os-miR396c* resulted in similar leaf phenotype and down-regulated *AsGRFs*, implying that *miR396*-*GRF* pathway is functionally conserved in different plant species.

The role of GRFs in regulating leaf development has been well characterized in *Arabidopsis*. The quadruple mutant *atgrf1*/*2*/*3*/*4* exhibits smaller leaf size because of the reduced cell size and cell number^40^. *AtGRF1* and *AtGRF2* control cell expansion, while *AtGRF3* and *AtGRF4* regulate cell proliferation^40^. In this study, we compared the number of leaf epidermis cells per unit leaf area between WT and TG plants. The result shows that TG plants have significantly fewer leaf epidermis cells per unit area than that of WT controls, which also implies larger cell size in TG plants. Additionally, RT-PCR analysis shows that *As1053*, a putative gene encoding anaphase-promoting complex subunit, which is involved in cell proliferation, is down-regulated in miR396 TG plants. Thus, it is likely that *miR396c*/*GRF* module control leaf area through regulating cell proliferation instead of cell expansion. This is further supported by the GO-enrichment analysis, in which the GO terms of ‘DNA replication’, ‘cell division’, ‘regulation of cell cycle’, and ‘mitotic nuclear division’ were significantly enriched in the down-regulated genes.

In grasses, tillering plays an essential role in shoot architecture and grain yield. In this study, tillering differences were observed between WT and miR396 TG creeping bentgrass. TG plants accumulate less shoot biomass, and have fewer tillers with reduced tiller length due to shorter internodes, suggesting that miR396 is implicated in the regulation of tillering. The development of grass tillers is determined by shoot apical meristems (SAMs), since all aerial organs are produced from SAMs. Previous studies show that *KNOTTED1*-*LIKE HOMEOBOX* (*KNOX*) transcription factor genes are key controllers on SAM formation and maintenance^41,42^. Recently, GRFs have been reported to regulate the expression of KNOX family gene *OsKN2* in rice through interacting with the promoter of *OsKN2*^43^. The similar interactions between GRF and *KNOX* have also been reported in *Arabidopsis* and barley^43,44^. Therefore, we speculate that the altered tillering in TG creeping bentgrass may be mediated through the *miR396*-*GRF*-*KNOX* regulatory module.

MiR396 is also involved in the modulation of root development. TG *Medicago truncatula* overexpressing miR396b exhibits reduced root dry weight, while MIM396, the inactive form of miR396 TG line displays significantly increased root dry weight compared with WT controls^45^. Further analysis indicates that miR396 modulates root growth through restricting the activity of root cell division^43^. In this study, the phenotype of reduced root dry weight is also observed in miR396 TG creeping bentgrass, indicating the conserved function of miR396-mediated root development in both monocots and dicots.

### MiR396-mediated regulatory network in plant response to salt stress

Plant responses to high salinity conditions have been extensively studied at the molecular level. The products of high salinity-responsive genes include downstream functional proteins and upstream regulatory proteins. In this study, *AsSOS1*, encoding the downstream functional Na^+^/H^+^ antiporter, was up-regulated in miR396 TG plants. Under salinity stress, SOS1 functions in maintaining ion homeostasis. Previous studies show that overexpression of *SOS1* leads to enhanced salt tolerance in TG *Arabidopsis*, tobacco, and *Chrysanthemum crassum*^11,46,47^. It is plausible that the enhanced salt tolerance in TG creeping bentgrass may result from miR396-mediated positive regulation of *AsSOS1*.

In addition, we also investigated the potential role other important Na^+^ transporters, NHX and HKT may play in miR396-mediated plant stress tolerance. NHX transporters are one of the best-characterized Na^+^ transporters, which alleviate excess Na^+^ through mediating intracellular Na^+^/H^+^ and K^+^/H^+^ antiport to compartmentalize Na^+^ into the vacuoles^5^. To examine the possible involvement of NHX in miR396-mediated plant response to salt stress in creeping bentgrass, a rice NHX gene *OsNHX1* was used to blast against the creeping bentgrass RNA-seq data to identify and clone the *AsNHX* gene. Real-time PCR analysis of *AsNHX* expression revealed no significant difference between miR396 TG and WT control plants (Figure S6a), suggesting that NHX may not be involved in miR396-mediated plant salt stress tolerance in miR396 TG creeping bentgrass. Our results indicate that the enhanced salt tolerance in miR396 TG plants is associated with reduced Na^+^ uptake, which suggests the adaptive mechanism of Na^+^ exclusion instead of Na^+^ compartmentalization into the vacuole in TG plants.

HKT transporters exhibit species-specific function due to their different selectivity for Na^+^ and K^+^. In wheat, TaHKT2;1 showed Na^+^/K^+^ co-transport activity at high Na^+^ as compared to K^+^ in extracellular compartment^48,49^. In T. *salsuginea*, a plant closely related to *Arabidopsis*, TsHKT1;2 showed higher selectivity for K^+^ over Na^+^^50^. In *Arabidopsis*, AtHKT1;1 showed a strong Na^+^ selective transport activity. AtHKT1 may participate in the Na^+^ uptake and Na^+^ circulation in plant body^51^. One of the rice HKTs exhibited the similar function^52^. To study the possible role of HKT in miR396-mediated plant salt tolerance in creeping bentgrass, a rice gene *OsHKT1* was used to blast against RNA-seq data in creeping bentgrass and identify *AsHKT1* for further study. Quantitative RT-PCR analysis of *AsHKT1* revealed a significantly reduced gene expression in miR396 TG creeping bentgrass compared with WT controls (Figure S6b), suggesting its possible involvement in the miR396-mediated plant salt stress response pathway. It is plausible that AsHKT1 may have higher selectivity for Na^+^ than K^+^. Overexpression of miR396 negatively impacts *AsHKT1* expression, leading to a reduced accumulation of Na^+^ in TG plant cells under salt stress conditions. The exact role *AsHKT1* plays in miR396-mediated salt stress response in creeping bentgrass remains to be further explored in the future.

Besides ionic stress, high salinity leads to the production of ROS, which damages plant cells and tissues through interacting with key macromolecules and metabolites. Among functional proteins, antioxidant enzymes protect plants from oxidative stress caused by ROS accumulation. In this study, significantly enriched GO terms of ‘oxidation–reduction process’, ‘H_2_O_2_ catabolic process’, ‘response to oxidative stress’, ‘oxidoreductase activity’, and ‘peroxidase activity’ imply that miR396 is crucial in plant response to oxidative stress.

Upstream regulatory proteins involved in high salinity responses include TFs, protein kinases, and phosphatases. The GO-enrichment analysis shows that ‘regulation of transcription’ and ‘regulation of kinase activity’ are significantly enriched in the down-regulated genes (Fig. S4b). Further RT-PCR analysis shows that genes encoding TFs from WRKY, MADS, and AP2 families are induced or down-regulated in miR396 TG plants (Fig. 6). TFs from WRKY family positively regulate salt and other environmental stress tolerance in a variety of plant species^53,54^. MADS and AP2 family TFs are also largely involved in plant abiotic stress resistance^55,56^. In addition to salt stress response, significantly enriched DEGs also participate in the biological processes of response to heat, cold, drought, heavy metal, wounding, high light intensity, nutrient deficiency stresses, and defense response to bacterium, virus, and fungus. Therefore, the result suggests that miR396, in a broad sense, may be actively involved in multiple environmental stress responses through modulating both functional and regulatory proteins.

MiR396 exerts its function at post-transcriptional and post-translational levels to regulate its targets, which encode TFs of GRF family and some species-specific TFs (e.g. bHLH79 in Medicago, SVP in *Arabidopsis*)^45,57^. These TFs will further regulate their direct targets, including TFs or other regulatory and functional proteins (e.g. AtGRF7 targets TF gene *DREB2A* in *Arabidopsis*, OsGRF3 and OsGRF10 target TF gene *OsKN2* in rice, OsGRF6 and OsGRF10 target protein kinase gene *OsCR4* and demethylase gene *OsJMJ706*)^39,58,59^. Therefore, miR396 serves as an important master regulator to integrate various regulatory elements to help plants cope with salt and other environmental stresses.

### Participation of GRFs in plant abiotic stress responses

In *Arabidopsis*, one of the miR396 target genes *AtGRF7* functions as a repressor of stress-responsive genes in plant abiotic stress response^59^. Under non-stressed conditions, *AtGRF7* overexpression lines and *atgrf7* mutants showed similar phenotype to WT controls. Interestingly, *AtGRF7* knock out mutants exhibited salt and drought tolerance in comparison with WT controls and *AtGRF7* overexpression lines. Further study showed that *AtGRF7* suppressed *DREB2A* (*DEHYDRATION-RESPONSIVE ELEMENT BINGING PROTEIN2A*) expression through biding to its promoter^59^. In addition, analysis of the T-DNA insertion mutant, atgrf7-1 revealed a large number of up-regulated genes that are involved in stress and abscisic acid responses, including *DREB2A*^59^. In our RNA-seq results, expression of *AsDREB2A* is also elevated in miR396 TG creeping bentgrass compared to wild type controls, which suggests that miR396-GRF7-DREB2A signaling pathway might play an important role contributing to the enhanced salt tolerance in TGs.

**Fig. 7:**
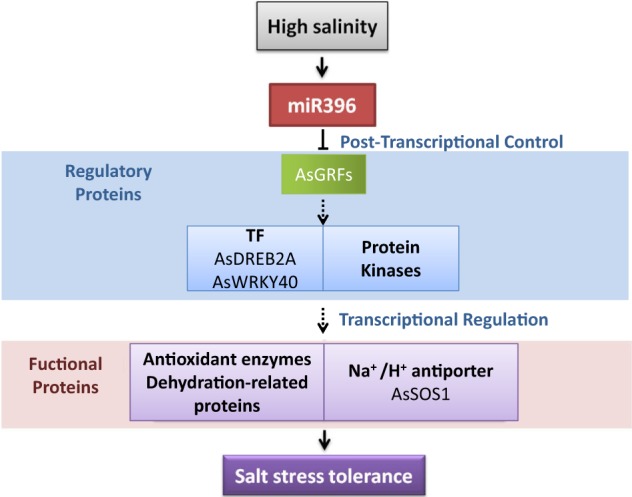
Hypothetical model of the signaling pathway of miR396-mediated salt stress tolerance

Taken together, the data obtained in our study suggest a potential signaling pathway of miR396-mediated salt tolerance in creeping bentgrass (Fig. 7). In response to high salinity, miR396 is induced to post-transcriptionally control the regulatory proteins of protein kinases and TFs, such as GRFs, DREB2A, and WRKY40. Next, these regulatory proteins activate downstream salinity responsive genes, such as Na^*+*^/H^+^ antiporter, *AsSOS1*; detoxification enzyme genes to protect plant cells from ionic stress and oxidative stress caused by high salinity stress; and DREB2A target genes to protect macromolecules from dehydration. This study provides insight into miR396-mediated salt tolerance and allows deciphering the role of miRNAs in the complex regulatory network in plant response to environmental stress.

## Supplementary information

120418_Revised miR396 for stress supplementary data.docx

120418_Marked-up version_Revised miR396 for stress manuscript.doc
