# Supplementary material for: MicroRNA396-mediated alteration in plant development and salinity stress response in creeping bentgrass
Source: Hortic Res. 2019 May 1;6:48. doi: 10.1038/s41438-019-0130-x (PMC6491569; doi:10.1038/s41438-019-0130-x)
Supplement: Supplementary file 1 — 120418_Revised miR396 for stress supplementary data.docx [file 41438_2019_130_MOESM1_ESM.docx]

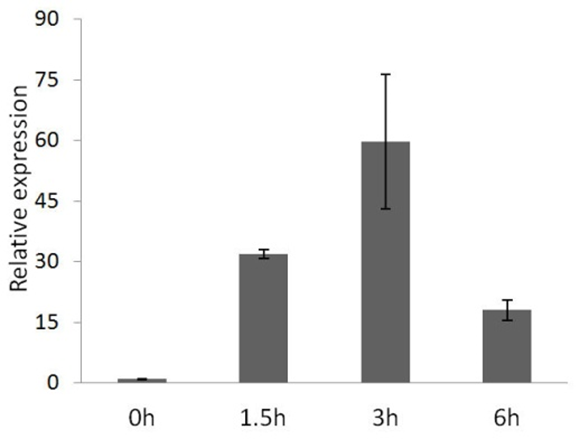


**Figure S1.** Expression profiles of miR396c in response to salt stress. Wild type (WT) creeping bentgrass plants were treated by 250 mM NaCl application. Leaf samples at 0, 1.5, 3, and 6 h after treatment were collected for analyzing relative expression levels of mature miR396 via stem-loop RT-qPCR. The relative changes in gene expression were calculated based on the 2^−∆∆CT^ method. *UBIQUITIN5* (*AsUBQ5*) was used as a reference gene. Data are presented as means of three technical replicates, and error bars represent ±SE.


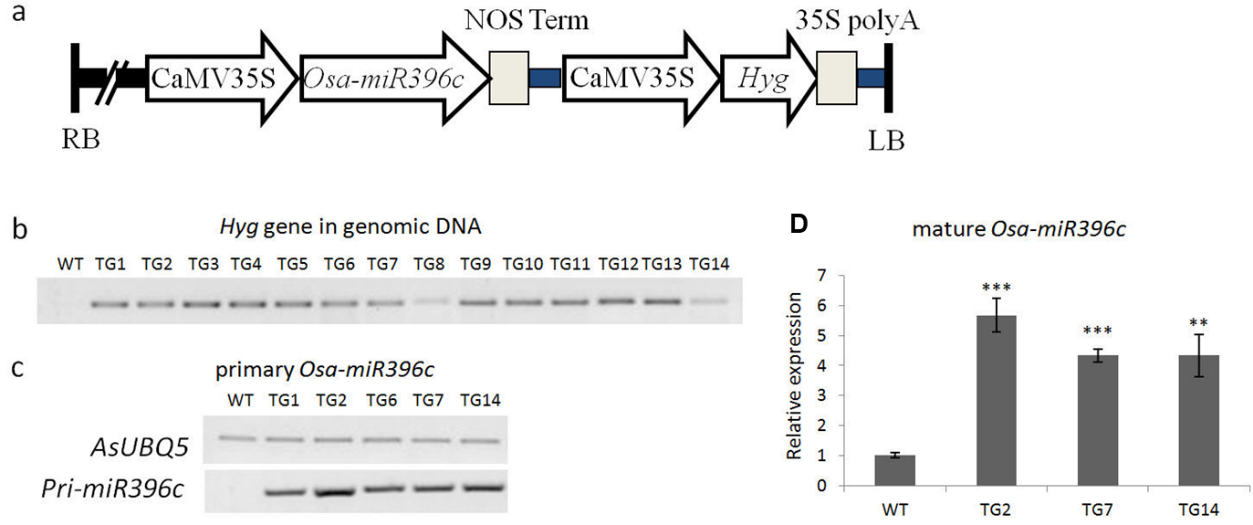


**Figure S2.** Generation of transgenic creeping bentgrass constitutively expressing *Osa-miR396c*. (a) *Osa-miR396c* gene constitutive expression construct, p35S-*Osa-miR396c*/p35S-*Hyg*. CaMV35S: Cauliflower Mosaic Virus 35S promoter; *Hyg*: the hygromycin resistance gene; RB: right border of T-DNA; LB: left border of T-DNA. (b) Examples of PCR analysis to amplify *Hyg* gene using genomic DNA of WT and transgenic (TG) plants. (c) Semi-quantitative RT-PCR analysis to compare the expression levels of primary *Osa-miR396c* in WT and TG plants. (d) Stem-loop RT-qPCR analysis to detect the expression of mature *Osa-miR396c* in TG and WT plants. The relative changes in gene expression were calculated based on the 2^−∆∆CT^ method. *AsUBQ5* was used as an endogenous control. Data are presented as means of three technical replicates, and error bars represent ± SE. Asterisks (** or ***) indicate a significant difference of expression levels between WT and each transgenic line at *P* < 0.01 or 0.001 by Student’s *t-test*.


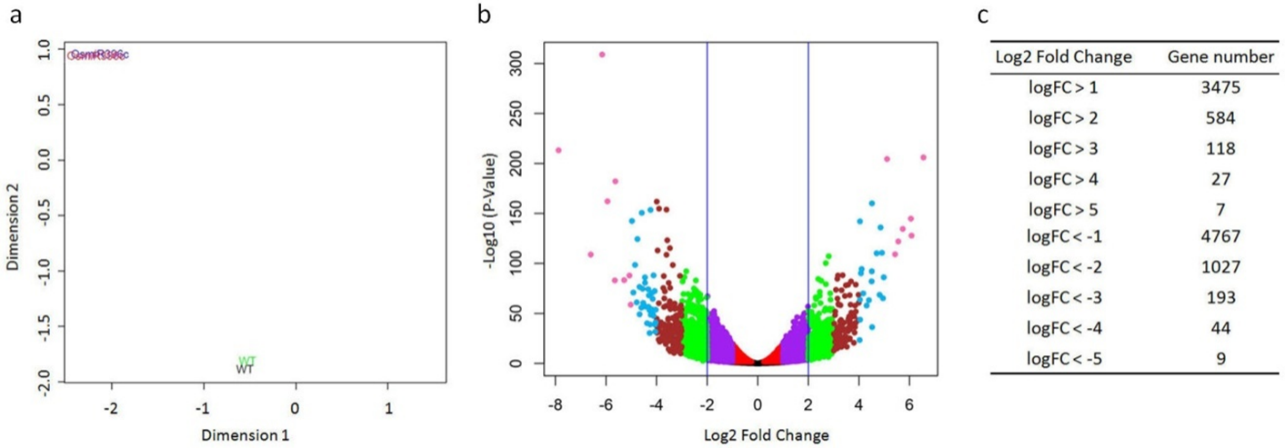


**Figure S3.** Differential expression of genes in TG vs. WT plants. (a) MDS plot of WT and Osa-miR396c TG RNA-seq samples with two biological replicates. (b) Volcano plot shows log2 FC of TG vs. WT data sets at LD non-stressed conditions. (c) A table shows log2 FC values and corresponding number of genes.


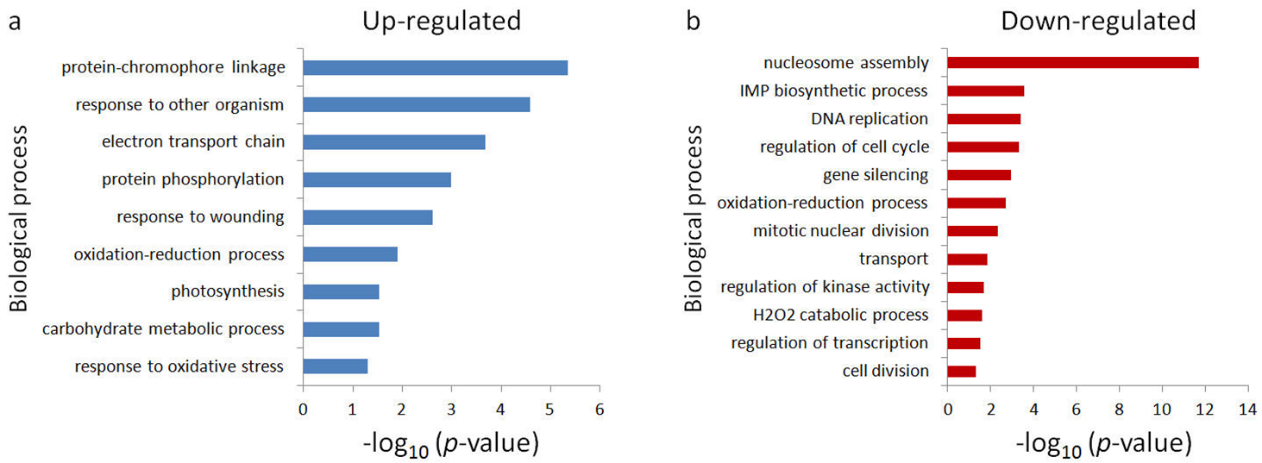


**Figure S4.** GO enrichment analysis. Significantly enriched GO terms show biological processes of (a) up-regulated (log_2_ FC > 2, over-represented *p*-value < 0.05) and (b) down-regulated (log_2_ FC < -2, over-represented *p*-value < 0.05) transcripts in TG vs. WT.


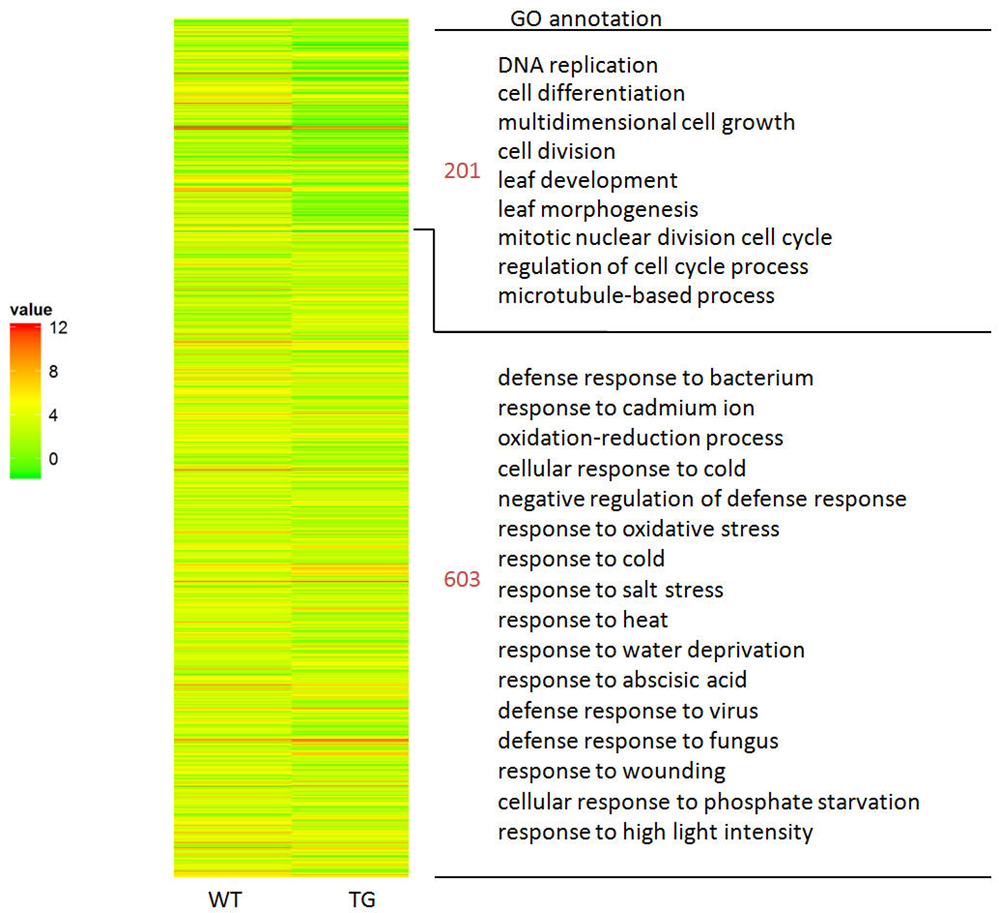


**Figure S5.** Differential expression of leaf development- and environmental stress response-related transcripts. Significantly enriched genes (over-represented p-value < 0.05) from each group were selected to generate the heatmap. Right to the heatmap shows the number of significantly enriched genes and GO terms. The color gradient shows the log2-transformation of the read count value.

**Figure S6.** Expression profiles of Na^+^ transporters *AsNHX1* (a) and *AsHKT1* (b)in WT and transgenic creeping bentgrass under normal growth conditions. The relative changes in gene expression were calculated based on the 2^−∆∆CT^ method. *UBIQUITIN5* (*AsUBQ5*) was used as a reference gene. Data are presented as means of three technical replicates, and error bars represent ±SE. Asterisks (***) indicate a significant difference of expression levels between WT and each transgenic line at *P* < 0.001 by Student’s *t-test*.
