# Supplementary material for: MicroRNA396-mediated alteration in plant development and salinity stress response in creeping bentgrass
Source: Hortic Res. 2019 May 1;6:48. doi: 10.1038/s41438-019-0130-x (PMC6491569; doi:10.1038/s41438-019-0130-x)
Supplement: Supplementary file 2 — 120418_Marked-up version_Revised miR396 for stress manuscript.doc [file 41438_2019_130_MOESM2_ESM.docx]

**Supporting information**

**Supplementary Table 1** Primer sequences used in this study.

| **Name** | **Sequence (5' - 3')** | **Purpose** |
| --- | --- | --- |
| ***Osa-miR396c* F** | TCTAGATTTTAACCCATCCAATGCCC | Insert restriction site *XbaI* |
| ***Osa-miR396c* R** | GTCGACCTCTCTCTCTCTCTCTGCCTG | Insert restriction site *SalI* |
| ***Hyg* F** | TACACAGGCCATCGGTCCAGA | PCR for genomic DNA of transgenic plants |
| ***Hyg* R** | TAGGAGGGCGTGGATATGTC |  |
| ***miR396c* stem-loop RT** | GTCTCCTCTGGTGCAGGGTCCGAGGTATTCGCACCAGAGGAGACAAGTTC | For reverse transcript of *miR396* |
| ***AsUBQ* q-F** | GGCGTCATCGACCTTGTAGA | For q-PCR of *AsUBQ* |
| ***AsUBQ* q-R** | GACAACGTCAAGGCCAAGAT |  |
| ***miR396* q-F** | GCGGTTCCACAGCTT TCTT | For q-PCR of *miR396* |
| ***miR396* q-R** | TGGTGCAGGGTCCGAGGTATT |  |
| ***AsGRF3* q-F** | CAAGAAAGCCTGTGGAAACG | q-PCR for mRNA of *AsGRF3* |
| ***AsGRF3* q-R** | AGCGAGTGGTTCTGGAAAG |  |
| ***AsGRF4* q-F** | ACTACCGCCTCTTCCCC | q-PCR for mRNA of *AsGRF4* |
| ***AsGRF4* q-R** | CATTGCTACATGCTGAGAACG |  |
| ***AsGRF5* q-F** | GTTAAAACCAATGGCCTGTCTC | q-PCR for mRNA of *AsGRF5* |
| ***AsGRF5* q-R** | TGCTCCAGTCAAGAAACTCAG |  |
| ***AsGRF6* q-F** | CCAGGCTGGAGAGTGTTTATG | q-PCR for mRNA of *AsGRF6* |
| ***AsGRF6* q-R** | GCGGATGCTCGGATGATTTA |  |
| ***AsSOS1* q-F** | CATCAACCTGATCGAGACCATC | q-PCR for mRNA of *AsSOS1* |
| ***AsSOS1* q-R** | CCATGAGGATGGAGGAGGT |  |
| ***AsNHX1* q-F** | CATATACCTCCTGCCTCCAATC | q-PCR for mRNA of *AsNHX1* |
| ***AsNHX1* q-R** | GTCCCAACAGCACCAAATAATG |  |
| ***AsHKT1* q-F** | TCCTGGGAAAGCTGACAAAG | q-PCR for mRNA of *AsHKT1* |
| ***AsHKT1* q-R** | GACGACTGTTGAGGAGAGAAAC |  |
| **As1053-F** | ATGTCCGCCAGATCAAGATATAC | PCR for mRNA of As1053 |
| **As1053-R** | CGACCCAACTAGGTTCATCAG |  |
| **As3793-F** | CCGAGGAGGACACCTGTA | PCR for mRNA of As3793 |
| **As3793-R** | CTCCAGAGCGCGAAATCA |  |
| **As37603-F** | GGAGAGCTTAGAAGCCCTAAAG | PCR for mRNA of As37603 |
| **As37603-R** | GGGTCTTCAGGTAGCATCATC |  |
| **As40994-F** | TCTACCAGTTCTCCTCATCCTC | PCR for mRNA of As40994 |
| **As40994-R** | CGCAGAGTTCATCCACCAATA |  |
| **As71896-F** | CGCCATTGATACCCGTCTTT | PCR for mRNA of As71896 |
| **As71896-R** | GAGGCAGAGCATGTTGATGT |  |
